# Unlocking the Conformational Changes of P2Y_12_: Exploring an Acridinone Compound’s Effect on Receptor Activity and Conformation

**DOI:** 10.3390/molecules28093878

**Published:** 2023-05-04

**Authors:** Belal O. Al-Najjar, Fadi G. Saqallah

**Affiliations:** 1 Department of Pharmaceutical Sciences, Faculty of Pharmacy, Al-Ahliyya Amman University, Amman 19328, Jordan; fadighassan92@gmail.com; 2 Diagnostic Research Centre, Al-Ahliyya Amman University, Amman 19328, Jordan

**Keywords:** P2Y_12_, molecular dynamics, MM-PBSA, P2Y_12_ antagonists

## Abstract

The P2Y_12_ receptor is an important member of the purinergic receptor family, known for its critical role in platelet activation and thrombosis. In our previously published study, the acridinone analogue NSC618159 was identified as a potent antagonist of P2Y_12_. In this work, we investigate the conformational changes in P2Y_12_ when bound to NSC618159 using molecular dynamics simulations on the receptor’s active and inactive forms (4PXZ and 4NTJ, respectively). It was observed that it took the systems about 7 ns and 12 ns to stabilise when NSC618159 was in complex with the active and inactive forms of P2Y_12_, respectively. Additionally, the binding pocket of the crystal structure 4PXZ expanded from 172.34 Å^3^ to an average of 661.55 Å^3^ when bound to NSC618159, with a maximum pocket volume of 820.49 Å^3^. This expansion was attributed to the pulled away transmembrane (TM) helices and the adoption of a more open conformation by extracellular loop 2 (EL2). In contrast, 4NTJ’s pocket volume was mostly consistent and had an average of 1203.82 Å^3^. Moreover, the RMSF profile of the NSC618159-4PXZ complex showed that residues of TM-I and TM-VII had similar fluctuations to the 4NTJ crystal structure, representing the inactive form of P2Y_12_. Finally, the energy components and binding affinities of NSC618159 towards the active and inactive forms of P2Y_12_ were predicted using the MM-PBSA approach. According to the results, the binding affinity of NSC618159 towards both active (4PXZ) and inactive (4NTJ) forms of P2Y_12_ was found to be almost identical, with values of −43.52 and −41.68 kcal/mol, respectively. In conclusion, our findings provide new insights into the conformational changes of P2Y_12_ upon binding to NSC618159 and may have implications for the development of new P2Y_12_ antagonists with enhanced potency and specificity.

## 1. Introduction

The P2Y_12_ receptor is an attractive target for anti-platelet therapies aimed at treating various thrombotic diseases. The P2Y_12_ protein is located on the surface of platelets and plays a crucial role in amplifying the response of P2Y_1_, which is responsible for platelet aggregation and the formation of blood clots via ADP-induced platelet activation [1,2]. The P2Y_12_ protein is composed of 342 amino acid residues and has two potential *N*-linked glycosylation sites at its amino terminus. Its structure includes seven hydrophobic transmembrane regions (TM-I through TM-VII), which are connected by three extracellular loops (EL1–3) and three intracellular loops (IL1–3). The binding of the agonist 2-methylthio-ADP leads to conformational changes in the extracellular loops, resulting in an inward movement of the transmembrane helices, whereas the antagonist AZD1283 prevents the inward movement of the transmembrane helices and blocks the formation of the disulphide bond between the TM-III helix and EL2 [1,2].

The study of the conformational behaviour of the P2Y_12_ receptor in response to binding with agonists and antagonists is of significant scientific interest. A thorough understanding of the conformational changes that occur in receptors following the binding of different ligands is critical to uncovering the mechanisms of receptor activation and inhibition, as well as to developing more effective drugs. Therefore, exploring the conformational differences between receptor–agonist and receptor–antagonist complexes is crucial to gaining a deeper understanding of the molecular interactions that underlie drug design for improved efficacy [3,4,5].

**Figure 1:**
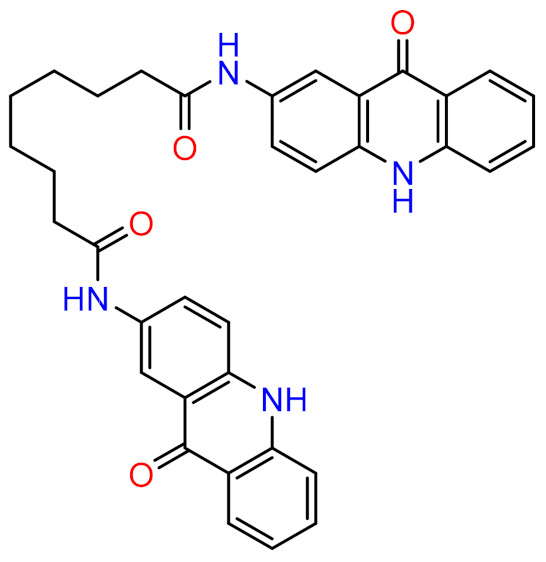
2D chemical structure of the potential P2Y_12_ inhibitor, NSC618159.

The current study aims to elucidate the mechanism of inhibition between P2Y_12_ and the previously reported inhibitor (NSC618159) [4], using molecular dynamics simulations and MM-PBSA calculations (Figure 1). By conducting a molecular modelling investigation, this study aims to gain insights into the structural dynamics of the P2Y_12_ protein and the potential mechanisms of inhibition that can inform the design of more effective drugs for thrombotic diseases.

Currently, developing novel biological applications is an active area of research in the scientific community. Compounds from marine sources [6], racemic synthetic compounds [7], and plant extraction [8,9,10] are popular sources for the discovery of novel bioactive molecules with therapeutic potential. Additionally, promoting neurogenesis and the proliferation of endogenous neural stem cells is an emerging strategy for the treatment of neurodegenerative diseases and brain injuries [11]. These approaches hold significant promise for the development of new therapeutics for a range of diseases and conditions.

This study was also encouraged by the drug-likeness properties of the acridinone compound NSC618159, which has a molecular weight of 572.65 Da, hydrogen bond donor (HBD) count of 4, hydrogen bond acceptor (HBA) count of 8, a LogP value of 3.98, 10 rotatable bonds, and a polar surface area (PSA) of 116 Å^2^ [12]. Hence, NSC618159 violates Lipinski’s rule for drug-likeness of orally given drugs by only one property, that is, the molecular weight being more than 500 Da. However, and according to recent literature, this rule for the molecular weight is not absolute and can, sometimes, be higher than the 500 Da cut-off. Notably, more than 200 compounds from Lipinski’s original publication, which were proceeded with in Phase II clinical trials, had molecular weights > 500 Da and approximately 170 compounds had molecular weights > 600 Da [13].

Interestingly, NSC618159 has fluorescent properties which are attributable to the acridinone ring systems. It is well documented that acridine orange has been employed and recognised in several medical fields, including microscopy, fluorescence guidance, and photo- and radio-dynamic therapy. Hence, the presence of the acridinone scaffold in an anti-platelet drug, i.e., NSC618159, would present several benefits for experimental studies. For example, the incorporation of such a fluorescent moiety facilitates the visualisation of the drug’s distribution and uptake in various tissues and cells when studied via in vivo or clinical studies. Moreover, the fluorescent probe can assist in evaluating the extent of platelet inhibition by the drug through the utilisation of fluorescence microscopy or flow cytometry [14].

## 2. Results

In theory, it is expected that P2Y_12_ becomes activated when it binds to an agonist. The seven transmembrane helices (TM-I through VII) of the protein would pull together, creating a narrow barrel-like binding pocket. In addition, the extracellular loop (EL2) between TM-IV and TM-V would fold inwards to close the binding pocket allowing Cys175 to interact with the agonist for the expression of the P2Y_12_ activity. On the contrary, if P2Y_12_ binds to an antagonist, the TM helices would expand, and EL2 would fold upwards to stop the protein expression [15].

**Figure 2:**
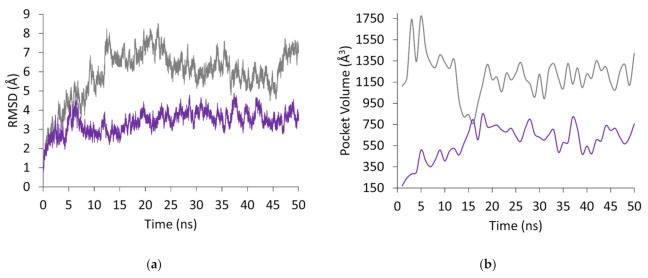

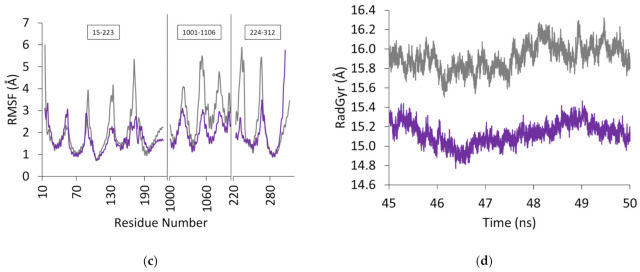
Trajectory analysis of the 50 ns simulation of NSC618159 in complex with P2Y12 crystal structures 4PXZ (purple) and 4NTJ (grey), showing the (**a**) root mean square deviation (RMSD), (**b**) pocket volume analysis, (**c**) root mean square fluctuation (RMSF), and (**d**) radius of gyration (RadGyr) of the non-hydrogen atoms at the binding pocket for the last 5 ns of the simulations.

In our investigation, and because NSC618159 was proven to be a potent antagonist of P2Y_12_ with a platelet reactivity index (%PRI) of 29.70% (±1.59) [2], it was employed in molecular dynamics simulations for 50 ns to study the effect of its binding to the crystal structure of P2Y_12_ in its active and inactive forms; i.e., 4PXZ and 4NTJ, respectively. Herein, RMSD analysis of NSC618159 in complex with the active form of P2Y_12_ (PDB ID: 4PXZ) shows that it took the complex about 7 ns to stabilise (Figure 2a), and the mean RMSD value for this system is 3.40 Å (±0.56). However, some minor inconsistencies can be seen around 37 and 42 ns to a maximum of 4.88 Å. These inconsistencies can be explained by the expansion of the binding pocket as indicated by its volume measurement (Figure 2b).

**Figure 3:**
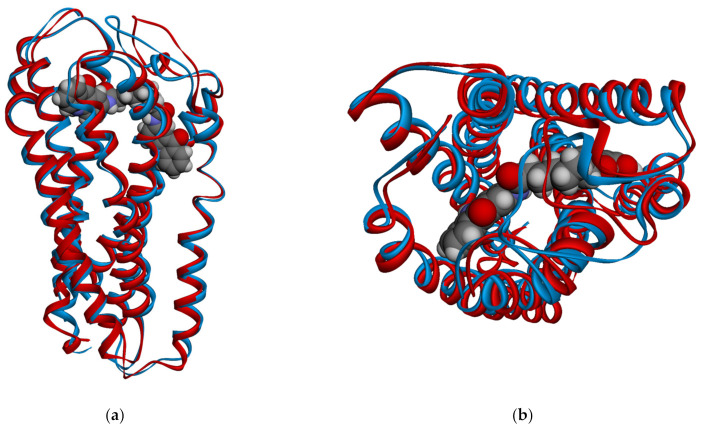
Snapshots at 36 ns (blue ribbon) and 37 ns (red ribbon) of NSC618159-4PXZ complex showing the expansion of the TM helices by 1.93 Å and the EL2 loop adopting an open conformation in (**a**) side view, and (**b**) top view.

Overall, the binding pocket of the crystal structure 4PXZ was seen to expand when bound to NSC618159 from 172.34 Å^3^ to an average of 661.55 Å^3^ (±93.38). The elevation in the pocket volume is believed to be stated after about 7 ns of the simulation, which agrees with the RMSD profile of the 4PXZ system. This expansion is attributed to the pulled away TM helices and the adoption of a more open conformation by EL2. Hence, between 36 and 37 ns, the TM helices and EL2 of 4PXZ were expanded by 1.93 Å (Figure 3), which allowed the pocket volume to reach a maximum of 820.49 Å^3^.

**Figure 4:**
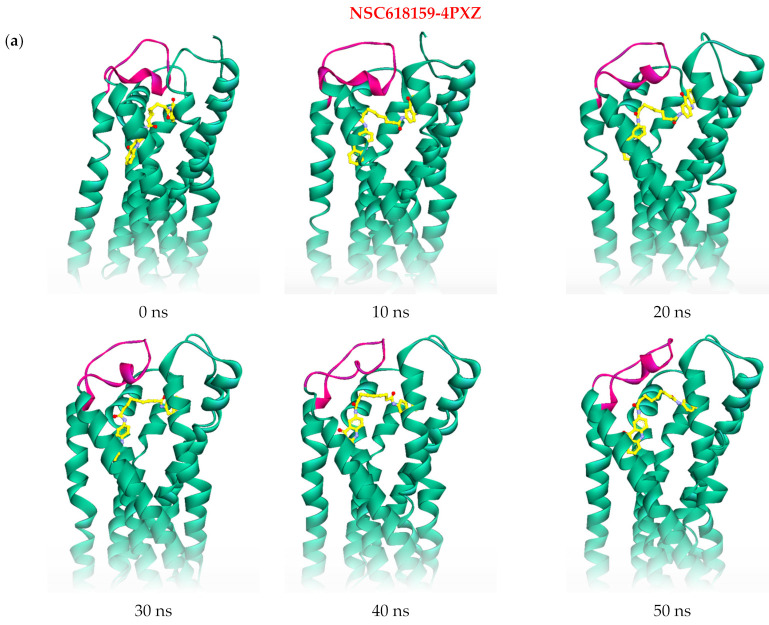

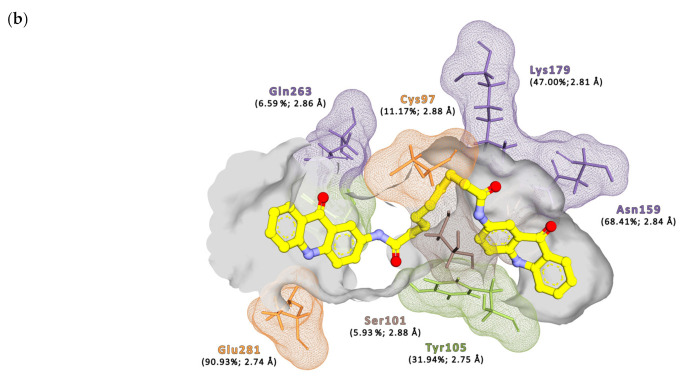
(**a**) Snapshots of the 50 ns molecular dynamics simulation of NSC618159 in complex with the active form of P2Y_12_ (4PXZ) illustrating the expansion of the receptor’s binding pocket and the outwards folding of EL2. (**b**) The key residues at the active site of P2Y_12_ active form (4PXZ) which interact through hydrogen bond interactions with NSC618159, along with their occupancies and interaction lengths.

A similar scenario can also be seen between 41 and 42 ns where the TM helices I and VII expanded by 0.54 and 0.59 Å, respectively. These findings can further be supported by the RMSF profile of NSC618159-4PXZ complex (Figure 2c), where residues Lys22-Thr42 of TM-I, and residues Thr271-Pro295 of TM-VII, had similar fluctuations to the 4NTJ crystal structure, which originally represent the inactive form of P2Y_12_. On the other hand, residues Asn164-Leu184, which form EL2, fluctuated, reaching about 2.62 Å trying to open the loop to cease the expression of P2Y_12_ activity. Nevertheless, the crystal structure 4PXZ, when in complex with NSC618159, was able to maintain its degree of rigidity indicated by its RadGyr profile during the last 5 ns of the simulation (Figure 2d), which was seen to undulate within less than 0.70 Å (between 14.77 and 15.46 Å) with a mean value of 15.13 Å (±0.11). The overall conformational changes in 4PXZ throughout the 50 ns simulation can be seen in Figure 4a.

Furthermore, hydrogen bond analysis of the 4PXZ system showed that NSC618159 was able to maintain four hydrogen bond interactions as a donor with Glu281 (90.93%) and Asn191 (7.98%) through the hydrogens of the acridinone rings nitrogens, with Cys97 (11.17%) through one of the amides’ nitrogens’ protons, and with Ser101 (5.93%) using the same proton of the amide’s nitrogen as in the case of Cys79. Hence, the disulphide interaction between Cys97 and Cys175 is impeded. Yet, NSC618159 was able to accept protons from various amino acids of P2Y_12_ to form seven more hydrogen bond interactions, especially with Asn159, Tyr105, Tyr259, Lys179, and Gln263 (Table 1 and Figure 4b).

On the contrary, the RMSD profile of NSC618159′s binding to the inactive form of P2Y_12_ (PDB ID: 4NTJ) showed that it took the complex an extended amount of time to stabilise, about 12 ns (Figure 2a). Herein, the mean RMSD value for this system after 12 ns is 6.42 Å (±0.72) with more fluctuations in comparison to the 4PXZ system. After stabilisation, the minimum RMSD value is 4.49 Å at around 44.42 ns, while the highest is 8.44 Å at 22.55 ns (Figure 2a). Such major variations in the RMSD profile are expected since the P2Y_12_ crystal structure 4NTJ was originally crystallised with the antagonist AZD1283. Hence, it took the complex some time to stabilise and adjust to the newly bound antagonist, NSC618159. These variations can further be explained by the binding pocket volume calculation of the 4NTJ complex where between 14 and 19 ns the binding pocket was seen to contract, reaching a minimum of 759.80 Å^3^. Thereafter, from 19 ns onwards, the binding pocket was seen to expand again to near its original volume (between 8 and 12 ns; 1334.69 Å^3^ (±53.37)) with an average of 1203.82 Å^3^ (±104.36) (Figure 2b).

Nevertheless, looking at the RMSF profile of the NSC618159-4NTJ complex, we can see higher fluctuations in a series of amino acid residues, including Gly88-Thr94 of TM-III, Thr127-Asn137 which form IL2 between TM-III and TM-IV, Thr210-Ala1001 of TM-V, Lys1019-Ala1024 of IL3 between TM-V and TM-VI, Lys1047-Phe239 which form the intracellular helices of P2Y_12_, Pro258-Phe268 of TM-VI (Figure 2c). It is noteworthy that residues Arg168-Ser176, which form EL2, fluctuated, reaching about 5.34 Å. This fluctuation in the EL2-forming residues can be explained by the time the complex needs to stabilise with NSC618159, where at the beginning the binding pocket of P2Y_12_ narrowed down before it expanded again to near its original volume, as explained earlier in the RMSD and pocket volume calculation.

**Figure 5:**
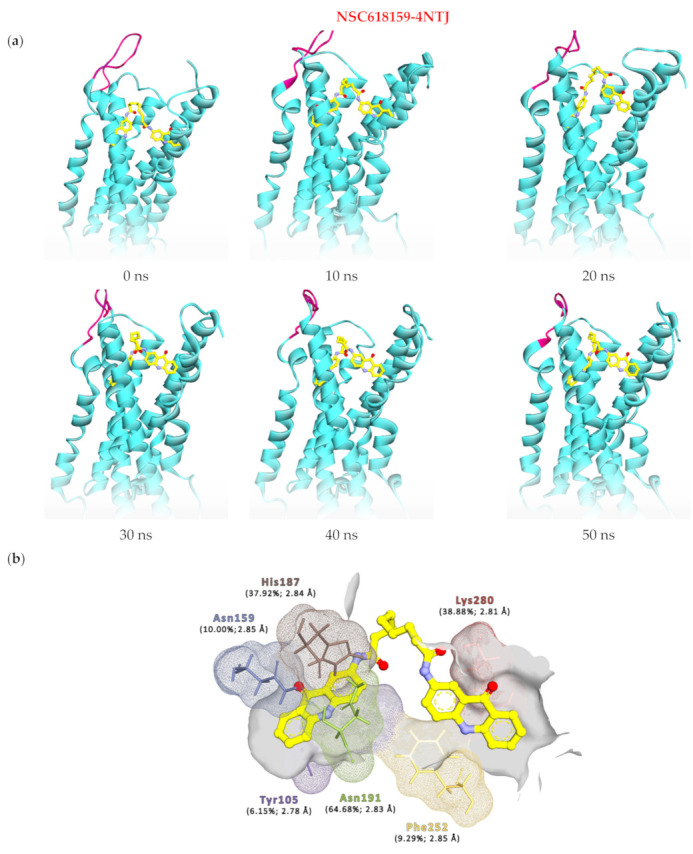
(**a**) Snapshots of the 50 ns molecular dynamics simulation of NSC618159 in complex with the inactive form of P2Y_12_ (4NTJ), illustrating the receptor’s binding pocket maintaining its volume while EL2 folds outwards of the pocket. (**b**) The key residues at the active site of P2Y_12_ inactive form (4NTJ), which interact through hydrogen bond interactions with NSC618159, together with their occupancies and interaction lengths.

Although the RadGyr calculation of the 4NTJ complex is slightly higher compared to the 4PXZ complex, it was noticed that the NSC618159-4NTJ complex was also able to maintain its degree of rigidity and overall compactness during the last 5 ns of the simulation (Figure 2d). This can be inferred from the 0.82 Å difference between the maximum and minimum RadGyr values (15.51 and 16.32 Å) with a mean value of 15.91 Å (±0.13). The overall conformational changes in 4NTJ throughout the 50 ns simulation can be seen in Figure 5a.

Moreover, hydrogen bond analysis of the 4NTJ system showed that NSC618159 was able to maintain two hydrogen bond interactions as a donor with Asn191 (64.68%) and Phe252 (9.29%) through the hydrogens of the acridinone rings nitrogens. Yet, NSC618159 was able to accept protons from various amino acids of P2Y_12_ to form six more hydrogen bond interactions, especially with the terminal primary amine group of Lys280 through the oxygen of the amide carbonyl of NSC618159 (cum. 38.88%), with His187 through the other amide’s carbonyl (37.92%), with Asn159 through the ketone carbonyl of one of the acridinone rings (10.0%), and with Tyr105 through the same amide carbonyl’s oxygen as in the case of Lys280 (6.15%) (Table 1 and Figure 5b).

Additionally, energy components of the interactions between NSC618159 in complex with 4PXZ and 4NTJ were calculated via MM-PBSA employing 50 frames from the last 5 ns of the simulations. Herein, NSC618159 was seen to express its binding mainly through non-polar interactions in both complexes with amino acids lining the binding pocket of P2Y_12_ (Table 2). Remarkably, there was no significant difference in the ΔG_bind_ values of both complexes of −43.52 and −41.68 kcal/mol, for the 4PXZ and 4NTJ complexes, respectively. This indicates that NSC618159 was able to change the conformational state of the active form of P2Y_12_, i.e., 4PXZ, and bind to it with a similar binding affinity to that of the inactive form of P2Y_12_, i.e., 4NTJ. Hence, NSC618159 is a true potential antagonist of P2Y_12_ which can block the expression of its activity efficiently.

## 3. Discussion

The current study investigated the effect of NSC618159, a potential potent antagonist of P2Y_12_, on the protein’s active and inactive forms through molecular dynamics simulations. The results showed that the binding pocket of the crystal structure 4PXZ expanded when bound to NSC618159, attributed to the pulled away TM helices I and VII and the adoption of a more open conformation by EL2 [15]. This study also revealed that NSC618159 was able to accept protons from various amino acids of P2Y_12_ to form numerous hydrogen bond interactions which are essential to antagonising the receptor’s activity.

The findings in this study are consistent with the theoretical expectation that P2Y_12_ becomes inactivated when it binds to an antagonist, expanding the binding pocket of the protein, and blocking the protein from expressing its activity. In addition, our study’s results are consistent with the findings in published literature on the binding of P2Y_12_ to other antagonists [15,16,17,18]. For instance, the binding of P2Y_12_ to ticagrelor, a commonly used P2Y_12_ antagonist, has been reported to cause the protein’s conformational changes, leading to the formation of a more open and flexible binding pocket [19]. Similarly, the binding of another P2Y_12_ antagonist, cangrelor, has been shown to induce significant structural changes in the protein, leading to the opening of the binding pocket [20].

Moreover, the outwards folding of EL2 away from the binding pocket of P2Y_12_ in the 4NTJ complex can easily explain the absence of any hydrogen bond interaction between NSC618159 and Cys79. It is worth noting that it has been reported that some amino acid residues, namely Arg256, Tyr259, and His253, located within the transmembrane region TM-6, are essential for the activity of P2Y_12_ [21]. In this study it was shown that NSC618159 was able to interact with Tyr259 in the case of the 4PXZ complex, while this type of hydrogen bond interaction was absent in the 4NTJ complex. This observation may be explained by the differences in the overall binding conformation or electrostatic environment of the two complexes which might affect the binding of NSC618159 to Tyr259. However, non-nucleotide antagonists were reported to share the presence of a hydrogen bond interaction with Asn159 and Lys280 [18]. This type of interaction with these amino acids can be observed to be very pronounced and with excellent occupancies in the case of both complexes, i.e., NSC618159-4PXZ and NSC618159-4NTJ.

## 4. Methods

**Figure 6:**
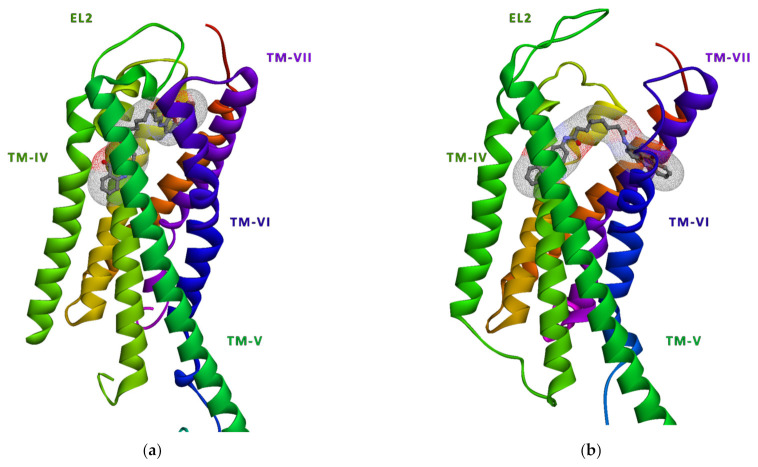
Initial binding conformations of NSC618159 to (**a**) the active form of P2Y_12_ (4PXZ) and (**b**) the inactive form of P2Y_12_ (4NTJ).

In our previous study dealing with the pharmacophore modelling and 3D-QSAR analyses of P2Y_12_, we came to identify NSC618159 as a potent antagonist [2]. Hence, the binding conformations of NSC618159 bound to the 3D crystal structures of P2Y_12_ in its active and inactive states (PDB IDs: 4PXZ and 4NTJ, respectively) were obtained by fitting the compound to the previously published pharmacophore (Figure 6). To further investigate the dynamic evolution of the binding conformation of NSC618159 at the binding pocket of P2Y_12_ in its active and inactive states, we conducted molecular dynamics simulations using AMBER 18 [22].

Briefly, 35 missing amino acid residues were added to the 4NTJ crystal structure using BIOVIA Discovery Studio 16.1 [23]. Additionally, the incomplete side chains of both crystal structures were fixed. The systems were then prepared using the LEaP program, assigning the ff14SB force field to the protein [24], and the AM1-BCC charge method [25,26] and the general AMBER force field (GAFF) to the ligand [27]. Both systems were neutralised with 18 and 19 chloride anions (Cl^-^) [28,29] for 4PXZ and 4NTJ, respectively. The protein–ligand complexes were further solvated in LEaP with a buffer of at least 10 Å in every axis, using a pre-equilibrated small box of 216 TIP3P water molecules [28,30]. The final volumes of the prepared systems were 10.653 × 10^5^ Å^3^ for the NSC618159-4PXZ system, and 11.332 × 10^5^ Å^3^ for the NSC618159-4NTJ system.

To minimise any possible steric clashes, steepest descent minimisation (5000 steps) was applied, followed by conjugate gradient minimisation (5000 steps) with a cut-off value of 9.0 Å. Next, the systems were heated through a multistep process, gradually raising the temperature from 0° to 310° K using the canonical ensemble (NVT). The systems were then equilibrated for 3 ns using the isothermal–isobaric ensemble (NPT). Finally, production was executed for 50 ns with a Langevin thermostat collision frequency γ at 1 ps^−1^.

Trajectories of the simulations were analysed using CPPTRAJ for their root mean square deviation (RMSD), root mean square fluctuation (RMSF), radius of gyration (RadGyr) for the last 5 ns, the secondary structure content using the DSSP algorithm, and H-bond interactions [31]. Additionally, the change in the binding pocket volume was monitored using POVME 2.2.1 at every single nanosecond of the simulation [32]. Finally, 50 frames from the last 5 ns of the simulations were analysed for their bonded and non-bonded energy components using the MM-PBSA python module [33].

## 5. Conclusions

The present study shows that the binding of NSC618159 to P2Y_12_ induces significant structural changes in the protein, leading to the opening of the binding pocket. These findings are consistent with the theoretical expectations and support the literature’s findings on the binding of P2Y_12_ to other antagonists. Hence, our study’s results contribute to the understanding of the mechanism of action of P2Y_12_ antagonists and may have implications for the development of more effective drugs targeting this protein. Further studies are strongly encouraged to assess the anti-platelet pharmacological activity and potential toxicity of NSC618159 in an animal model. This step is a necessity to validate our findings and to further elucidate the reported structural and functional implications.

## Figures and Tables

**Table 1:** Hydrogen bond interaction analyses of NSC618159 when in complex with the two crystal structures of P2Y_12_, 4PXZ and 4NTJ, throughout the 50 ns simulation.

Complex	Acceptor	Donor	Occupancy (%)	Average Distance (Å)	Average Angle (Å)
4PXZ	GLU281@OE2	NSC159@H47/N9 *	90.93	2.74	154.18
	NSC159@O39 *	ASN159@HD22/ND2	68.41	2.84	159.27
	NSC159@O17 *	TYR105@HH/OH	31.94	2.75	160.15
	NSC159@O17 *	TYR259@HH/OH	17.84	2.77	162.74
	NSC159@O23 *	LYS179@HZ3/NZ	16.03	2.81	154.93
	NSC159@O23 *	LYS179@HZ2/NZ	15.73	2.81	155.21
	NSC159@O23 *	LYS179@HZ1/NZ	15.24	2.81	155.19
	CYS79@O	NSC159@H61/N24 *	11.17	2.88	161.19
	ASN191@OD1	NSC159@H66/N31 *	7.98	2.83	151.56
	NSC159@O17 *	GLN263@HE21/NE2	6.59	2.86	159.53
	SER101@O	NSC159@H61/N24 *	5.93	2.88	156.16
4NTJ	ASN191@OD1	NSC159@H66/N31 *	64.68	2.83	162.28
	NSC159@O17 *	HIS187@HE2/NE2	37.92	2.84	155.02
	NSC159@O23 *	LYS280@HZ2/NZ	14.10	2.81	154.99
	NSC159@O23 *	LYS280@HZ1/NZ	12.73	2.81	154.62
	NSC159@O23 *	LYS280@HZ3/NZ	12.05	2.81	154.49
	NSC159@O39 *	ASN159@HD22/ND2	10.00	2.85	159.42
	PHE252@O	NSC159@H47/N9 *	9.29	2.85	159.95
	NSC159@O23 *	TYR105@HH/OH	6.15	2.78	159.14

* The mentioned N, O, and H atoms of NSC618159 as hydrogen bond acceptors and donors are annotated with their corresponding numbers in the 2D structure of the ligand provided alongside this footnote. 
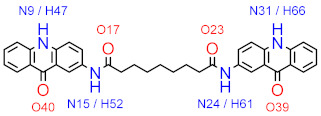
.

**Table 2:** Predicted energy components and binding affinities of NSC618159 towards the active and inactive forms of P2Y_12_ (4PXZ and 4NTJ, respectively) using MM-PBSA approach for the last 5 ns of the molecular dynamics simulations.

Energy Component (kcal/mol)	Energy Value (kcal/mol) per Complex	
	NSC618159-4PXZ	NSC618159-4NTJ
van der Waals Energy (ΔE_vdW_)	−71.783	−64.022
	±4.0481	±3.4897
Electrostatic Energy (ΔE_EL_)	−49.4273	−54.907
	±6.2724	±7.715
Polar Solvation Energy (ΔE_PB_)	84.6431	83.1979
	±8.5487	±9.1691
Non-Polar Solvation Energy (ΔE_NPOLAR_)	−6.9489	−5.9511
	±0.1474	±0.2037
Total Gas Phase Free Energy (ΔG_gas_)	−121.2103	−118.929
	±6.9472	±7.9284
Total Solvation Free Energy (ΔG_solv_)	77.6942	77.2468
	±8.5099	±9.1647
Total Energy (ΔG_bind_)	−43.5161	−41.6822
	±6.0448	±7.3535

## Data Availability

Not applicable.
